# Do the Testing Posture and the Grip Modality Influence the Shoulder Maximal Voluntary Isometric Contraction?

**DOI:** 10.3390/jfmk8020045

**Published:** 2023-04-14

**Authors:** Marco Bravi, Chiara Fossati, Arrigo Giombini, Elena Mannacio, Riccardo Borzuola, Rocco Papalia, Fabio Pigozzi, Andrea Macaluso

**Affiliations:** 1 Department of Movement, Human and Health Sciences, University of Rome “Foro Italico”, 00135 Rome, Italy; 2 Department of Physical and Rehabilitation Medicine, Università Campus Bio-Medico, 00128 Rome, Italy; 3 Centre for Exercise Science and Sports Medicine, University of Rome “Foro Italico”, 00135 Rome, Italy; 4 Department of Orthopaedic and Trauma Surgery, Università Campus Bio-Medico, 00128 Rome, Italy; 5 Villa Stuart Sport Clinic, FIFA Medical Centre of Excellence, 00135 Rome, Italy

**Keywords:** strength, MVIC, assessment, dynamometer, shoulder, posture, grip

## Abstract

Assessing and monitoring shoulder strength is extremely important during rehabilitation. A fixed dynamometer represents a valid and inexpensive assessment method. However, it has not been studied whether posture and grip modality influence shoulder muscle strength. The aim of this study was to compare shoulder strength values between sitting and standing positions and between the handle and cuff grip modalities. A total of 40 volunteers were divided into a posture (PG) and a handle-cuff group (HCG). Participants in the PG were asked to perform a maximum voluntary isometric contraction (MVIC) for shoulder flexion, extension, ab-adduction, and intra-extra rotation in standing and sitting positions. The HCG participants were tested in a standing position while holding a handle or with a cuff around their wrist. PG showed higher forces in the standing position for shoulder flexion (*p* = 0.009); internal rotation showed higher values in the sitting position (*p* = 0.003). ER/IR ratio was significantly higher in the standing position (*p* < 0.001). HCG showed higher significant forces during cuff modality in all positions and grip modalities, including the ER/IR ratio (*p* < 0.05). Different body positions and grip modalities influenced the assessment of shoulder strength as recorded by a fixed dynamometer; therefore, these factors should be carefully considered when carrying out a shoulder strength assessment, and we encourage the development of assessment guidelines to make future clinical trial results comparable.

## 1. Introduction

Strength assessment is widely used in rehabilitation settings, as it is frequently adopted to monitor recovery from an injury and represents an essential component of physical condition assessment. Muscle strength assessment becomes extremely important when it refers to the shoulder. This joint is substantially unstable due to poor bone congruence, and muscle forces play a significant role in contributing to the stability of the joint [1]. Furthermore, constant monitoring of the shoulder muscle strength allows personnel involved in managing shoulder injuries to adapt interventions and make decisions about the return to preinjury activities.

In clinical practice, there are different ways to quantify muscle strength of the upper limbs, which include manual muscle testing (MMT), isokinetic dynamometry (ID), handheld dynamometry (HHD), and fixed dynamometry (FD). MMT consists of testing muscles against the examiner’s resistance and grading the strength using a 0 to 5 points scale [2]. This method is largely used in clinical settings. However, it is susceptible to subjectivity, and, thus, the reliability level is still uncertain [3,4]. Furthermore, it is not highly sensitive in detecting strength differences, as it has been demonstrated that level 4 can be reached with just 20% of the maximum shoulder strength [5]. ID is considered the gold standard of muscle strength testing. Nevertheless, it is expensive and time-consuming, and the equipment requires a large dedicated space, resulting in an impractical clinical setting [6]. HHD is a less expensive method compared to ID. However, despite studies reporting acceptable inter-rater reliability, several factors, such as tester strength, joint position, force application, and stabilization of the patient, can influence the validity of this method [6,7,8]. FD can be a valid alternative to HHD as it is equipped with strain gauges like HHD; in addition, it entails an anchoring system by which it can be fixed to the wall, floor, or specific gym equipment (e.g., pulley cable systems), thus reducing the limitations of the HHD method [9,10].

The shoulder muscle strength assessment can be performed in different positions. Several studies used HHD and FD to assess shoulder muscle strength with the subject in a standing position, a sitting position, holding a handle, or with a loop around the wrist [11,12,13]. However, although studies on the reliability of dynamometric strength assessment have been published [14,15], to the best of the authors’ knowledge, no study has investigated the effects of the different testing postures (i.e., sitting position, standing position) and of the different fixing modalities through which the subject exerts force (i.e., holding a handle or with a cuff around the wrist) on maximum voluntary isometric contraction (MVIC). As a matter of fact, we believe that the different testing positions could influence MVIC. It is expected that in a seated position, the compensatory movements of the trunk and the shifting of body weight are less than in the standing position; moreover, the activation of the muscles of the lower extremities is weaker, resulting in a lower facilitation of upper limb muscles contraction [16]. Additionally, we hypothesized that the MVIC is greater when shoulder strength is tested by asking the participant to hold a handle, which represents a more stable grip that allows the development of greater strength. Therefore, the aim of this study was to compare shoulder strength values, recorded by an FD, between sitting and standing positions and between the handle and cuff grip modalities.

## 2. Materials and Methods

The study received the Ethical Committee of Campus Bio-Medico University of Rome approval (Prot. PAR 001.22(62.21)) and was held in accordance with the Declaration of Helsinki. All participants signed informed consent.

### 2.1. Participants

After a brief medical history interview to rule out any pathologies and/or the presence of shoulder pain, 40 healthy volunteers (sample of convenience based on the previous study by Saeterbakken et al. [17]) were enrolled according to the following inclusion criteria: (1) age > 18 years; (2) no history of shoulder pathologies (e.g., shoulder surgery, shoulder dislocation, rotator cuff pathology, or other macrotrauma); (3) no current pain. All participants were recruited from the University Campus Bio-Medico and Foro Italico University.

The following methods for shoulder strength testing were evaluated, as described in the literature [11,12,13]: (1) standing position with handle; (2) sitting position with handle; (3) standing position with a cuff around the wrist. Therefore, a within-participant design was used. Enrolled participants were divided into a posture group (PG) and a handle-cuff group (HCG) to study the effects of body position (sitting or standing) and grip modality on the shoulders’ maximal voluntary isometric contraction (MVIC). Participants’ demographics for each group are reported in Table 1.

### 2.2. Testing Procedures and Instruments

**Figure 1:**
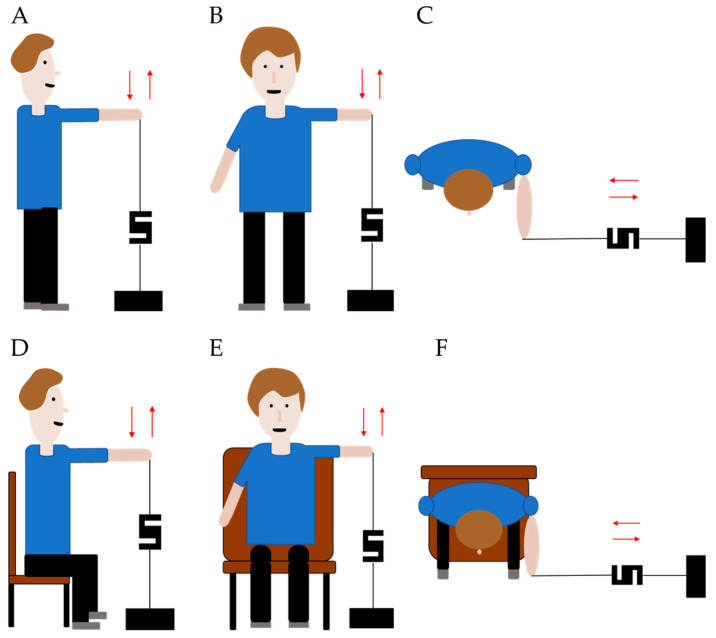
(**A**) Standing shoulder flexion and extension; (**B**) Standing shoulder abduction and adduction; (**C**) Standing shoulder internal and external rotation; (**D**) Sitting shoulder flexion and extension; (**E**) Sitting shoulder abduction and adduction; (**F**) Sitting shoulder internal and external rotation.

Shoulder muscle strength in flexion, extension, abduction, adduction, internal, and external rotation was measured as follows:

- Flexion (Flex) and extension (Ext). Shoulder at 90° of flexion, 0° of horizontal abduction, elbow extended, and forearm pronated (Figure 1A–D);
- Abduction (Abd) and adduction (Add). Shoulder at 90° flexion, 90° of horizontal abduction, forearm in neutral position (“full can position”) (Figure 1B–E);
- Internal (IR) and external rotation (ER). Shoulder in adduction and 45° of internal rotation of the humerus. Elbow flexed to 90°. Patients were asked to keep their elbows by their sides during the test (Figure 1C,F).

The MVIC was measured with the Chronojump Boscosystem^®^ (Barcelona, Spain) Force Sensor Kit. The force signal was amplified at 80 Hz using the dedicated amplifier and displayed in front of each participant on a PC Laptop. The force sensor was connected to a position-adjustable single pulley cable system. The pulley system was loaded with the maximum weights (up to 80 kg) while the subject performed the MVIC tasks.

**Figure 2:**
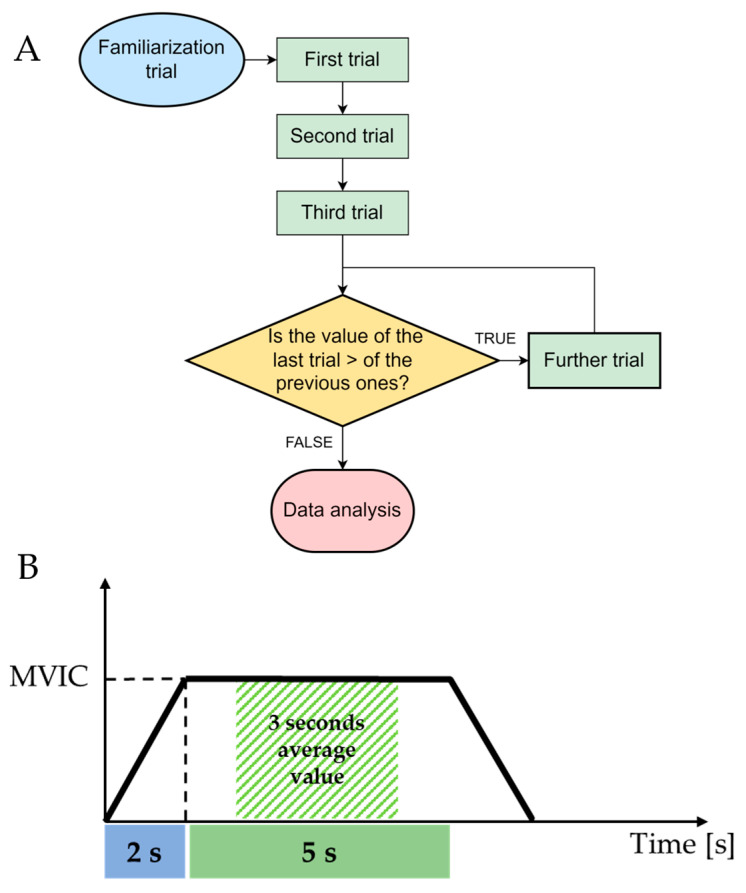
(**A**) Flow diagram of maximal voluntary isometric contraction (MVIC) measurements. (**B**) Detailed view of the experimental trial highlighting the 2 s used for building up the force gradually, the 5 s holding the MVIC, and the 3 s average values used for data analysis.

A minimum of 3 maximal attempts was performed for each task, interrupted by 3 min to recover from fatigue; subjects were asked to make a further attempt if the MVIC of their last trial exceeded that of previous trials (Figure 2). Participants were verbally encouraged to reach their maximal strength during each trial.

The same investigator performed the measurements and instructed each participant on the correct execution of the test, which consisted of building up the force gradually to a maximum effort (2 s), holding it for 5 s, and then returning to the rest position (Figure 2). A first trial test was performed to familiarize the investigator with the test and the instrumentation. The average value of the 3 s of maximum contraction was computed from each trial; the external rotation to internal rotation ratio (ER/IR) was calculated from the mean value recorded in ER and IR tests. Subsequently, the average value of the 3 trials was calculated and used for the analysis. To avoid any influence of fatigue on the results, the starting position of each test (i.e., sitting/standing or handle/cuff) was randomized.

### 2.3. Statistical Analysis

IBM SPSS Statistics version 23.0 software program (IBM Corp., Armonk, NY, USA, ABD) was used for the statistical analyses.

Since not all the variables were evenly distributed through Kolmogorov-Smirnov and Shapiro Wilks tests, non-parametric paired samples Wilcoxon test was used to compare the strength values between the two different testing postures (PG) and the two different fixing methods (HCG). Statistical significance was set at *p* < 0.05, according to a previous study [13].

## 3. Results

### 3.1. Posture Group

Mean values ± standard deviation recorded during the strength tests for PG are reported in Table 2. The results exhibited significantly higher forces in the standing position for shoulder flexion (*p* = 0.009). Internal rotation showed significantly higher strength values in the sitting position (*p* = 0.003). The ER/IR ratio was significantly higher in the standing position (*p* < 0.001).

### 3.2. Handle-Cuff Group

Mean values ± standard deviation recorded during the strength tests are reported in Table 3. There were significant differences between the two grip modalities, with significantly higher forces during cuff grip tests in flexion (*p* < 0.001), extension (*p* < 0.001), abduction (*p* = 0.001), adduction (*p* = 0.004), external rotation (*p* < 0.001), and internal rotation (*p* < 0.001). The ER/IR ratio was also significantly different (*p* = 0.010).

## 4. Discussion

The main finding of this study is that both body position and grip modality influenced the MVIC of shoulder muscles as measured by a fixed dynamometer.

Regarding body position, the PG showed significantly higher strength values in the standing position for shoulder flexion, while there were no significant differences for shoulder extension, abduction, adduction, and external rotation. However, it must be underlined that the differences in absolute values exhibited minimal variations, which were around 6 N for flexion, 3 N for abduction, 7 N for external rotation, 11 N for extension, and 15 N for adduction. These values are lower than the minimal detectable change (MDC) reported by Sciascia et al. [18]. A possible explanation for these results could be that the standing position compared to the sitting position allows for a more remote activation of the lower limbs and trunk muscles that contributes to an increase in the activation of the muscles of the upper limb, according to work by Ebben et al. [19]. This also agrees with the work of Saeterbakken et al. [17], in which higher strength values were reported in the standing position. In addition, the greater strength values in the standing position concerning the shoulder flexion task could be related to the activation of abdominal muscles. In fact, an anticipatory activation of the abdominal muscles is necessary to increase trunk stability and allows for the generation of greater forces [20]. Furthermore, Urquhart et al. [21] demonstrated that when rapid shoulder flexion is performed, the activation of the abdominal muscles is generally delayed in a sitting position compared to a standing position, thus explaining the higher strength values recorded in the standing position.

In contrast, internal rotation values showed that the sitting position favors the development of significantly greater strength, with a sizeable mean difference of about 20 N, compared to the standing position. The differences recorded in external and internal rotations tests resulted in a significantly different ER/IR ratio between the two positions. This aspect is extremely important as the ER/IR ratio represents a useful value for estimating the quality of muscle balance, particularly in a clinical environment. Specifically, some authors indicated that when this value is less than 66% (optimal range 66 to 75% [22,23]), it seems to be strongly correlated with an increased risk of shoulder injury [24,25,26]. Therefore, since our results showed that position significantly influences the ER/IR ratio (74% in standing and 63% in sitting), the evaluation position should be carefully chosen to avoid misinterpretation of the ER/IR ratio, especially when this value is used as a criterion to guide clinical decisions regarding injury recovery.

The results of the HCG showed a different trend. As a matter of fact, in all tests, a significant increase in strength was recorded when the test was performed with a cuff around the wrist. Absolute strength values show a minimum mean difference of 16 N for the adduction test and a maximum mean difference of 35.5 N for the external rotation test. However, the ER/IR ratio, although showing a significant difference between the two modalities, changed by 7% (72% with the handle to 79% with the cuff). These results can be explained by the findings of Sporrong et al. [27,28], which showed that high demands on handgrip force affect the activity of shoulder muscles, adding further high loads on these muscles. Therefore, when the participants performed the tests with the cuff around the wrist, shoulder muscles might have been able to express more force since they were not engaged in the hand grip activity. Another possible explanation could be that closing the fingers around the handle might involve the activation of the long flexors of the fingers with a combined synergistic action of the wrist extensors muscles. These muscles have the task of counteracting the wrist flexion caused by the action of long flexors of the fingers [29]. Therefore, according to Mandalidis et al. [30], it is likely that when shoulder strength was assessed with the cuff around the wrist, no wrist flexor activity was required; hence, the wrist extensor muscles were able to transmit more forces, via myotendinous and myofascial pathways, to the proximal joints. This might have ensured greater stability to the elbow and shoulder joints, allowing the shoulder muscles to develop greater strength.

The findings of the present study indicate that both body position and grip modality impact force-generating capacity. Therefore, both factors should be carefully considered when carrying out a strength assessment consistent with the activities that the patient/athlete will have to perform. For example, it would be more appropriate that an overhead athlete whose sporting gesture requires handgrip forces, such as a baseball pitcher who grips the ball in his hand or a tennis player who grips his racket, should be evaluated in a standing position with the handle grip, while a swimmer who, on the contrary, does not need a high handgrip force in the sporting gesture could be assessed in a standing position with the cuff around the wrist. From a clinical point of view it is crucial to properly assess shoulder strength, as it has already been demonstrated that the imbalance of the internal shoulder rotators and external rotators represents a possible risk factor for shoulder dysfunction [31]. Furthermore, strength assessment plays a key role when dealing with overhead athletes, as preseason muscle strength assessment has proven to be an effective strategy for identifying athletes at higher risk of injury, thus enabling the implementation of appropriate injury prevention programs [24]. In this context, a proper IR and ER strengths assessment is also essential to develop injury prevention and rehabilitation programs [32]. Consequently, it is important to consider body position and fixation modality when assessing shoulder muscle strength.

## 5. Conclusions

This study shows that different body positions (sitting or standing) and grip modality (holding a handle or with a cuff around the wrist) influence the assessment of shoulder strength recorded by a fixed dynamometer. In particular, the ER/IR ratio appears to be strongly influenced by the body position. Therefore, the aforementioned aspects should be carefully considered during a shoulder strength assessment. Moreover, guidelines for shoulder strength assessments would be required to make clinical trial results comparable.

## Figures and Tables

**Table 1:** Participant demographics.

Variable	Posture Group (n = 20) ^1^	Handle-Cuff Group (n = 20) ^1^
Age [years]	24.95 ± 6.82	28.10 ± 8.62
Gender [m–f]	11–9	12–8
Height [m]	1.75 ± 0.01	1.72 ± 0.08
Body Mass [kg]	71.25 ± 15.53	72.10 ± 12.25
BMI [kg/m^2^]	22.80 ± 2.77	24.06 ± 2.98

^1^ Values are expressed as mean ± standard deviation.

**Table 2:** Shoulder strength mean measures, standard deviations, and related *p*-values for both groups.

Shoulder Strength Tests	Standing	Sitting	Mean Difference	*p*-Value ^1^
Flexion [N]	114.89 ± 50.78	108.80 ± 49.12	6.08 ± 10.95	0.009
Extension [N]	195.68 ± 80.94	184.27 ± 64.34	11.40 ± 27.86	0.073
Abduction [N]	107.78 ± 49.34	103.82 ± 47.66	3.95 ± 14.19	0.159
Adduction [N]	179.37 ± 74.68	164.17 ± 64.18	15.19 ± 26.92	0.079
External rotation [N]	117.57 ± 41.94	110.32 ± 38.80	7.24 ± 19.84	0.140
Internal rotation [N]	160.35 ± 58.97	179.47 ± 68.78	−19.11 ± 26.74	0.003
ER/IR [Ratio]	0.74 ± 0.12	0.63 ± 0.14	0.11 ± 0.11	<0.001

^1^ Wilcoxon signed-rank test.

**Table 3:** Shoulder strength mean measures, standard deviations, and related *p*-values for handle and cuff tests.

Shoulder Strength Tests	Handle	Cuff	Mean Difference	*p*-Value ^1^
Flexion [N]	128.26 ± 52.14	148.41 ± 55.57	−20.15 ± 12.05	<0.001
Extension [N]	208.85 ± 74.70	237.08 ± 84.38	−28.23 ± 21.59	<0.001
Abduction [N]	119.61 ± 50.86	138.50 ± 50.49	−18.88 ± 18.79	0.001
Adduction [N]	193.46 ± 60.39	209.81 ± 63.45	−16.34 ± 20.55	0.004
External rotation [N]	123.30 ± 33.03	158.82 ± 45.97	−35.52 ± 20.72	<0.001
Internal rotation [N]	170.39 ± 35.42	202.86 ± 58.14	−32.46 ± 29.62	<0.001
ER/IR [Ratio]	0.72 ± 0.10	0.79 ± 0.12	−0.07 ± 0.1	0.010

^1^ Wilcoxon signed-rank test.

## Data Availability

The data will be made available upon reasonable request to the corresponding author.
